# Unsupervised local center of mass based scoliosis spinal segmentation and Cobb angle measurement

**DOI:** 10.1371/journal.pone.0300685

**Published:** 2024-03-21

**Authors:** Mohamed Yacin Sikkandar, Maryam M. Alhashim, Ahmad Alassaf, Ibrahim AlMohimeed, Khalid Alhussaini, Adham Aleid, Murad J. Almutairi, Salem H. Alshammari, Yasser N. Asiri, S. Sabarunisha Begum

**Affiliations:** 1 Department of Medical Equipment Technology, College of Applied Medical Sciences, Majmaah University, Al Majmaah, Saudi Arabia; 2 Department of Radiology, College of Medicine, Imam Abdulrahman Bin Faisal University, Dammam, Saudi Arabia; 3 Department of Biomedical Technology, College of Applied Medical Sciences, King Saud University, Riyadh, Saudi Arabia; 4 Medical Imaging Services Center, King Fahad Specialist Hospital Dammam, Dammam, Saudi Arabia; 5 Department of Biotechnology, P.S.R. Engineering College, Sivakasi, India; Ningbo University, CHINA

## Abstract

Scoliosis is a medical condition in which a person’s spine has an abnormal curvature and Cobb angle is a measurement used to evaluate the severity of a spinal curvature. Presently, automatic Existing Cobb angle measurement techniques require huge dataset, time-consuming, and needs significant effort. So, it is important to develop an unsupervised method for the measurement of Cobb angle with good accuracy. In this work, an unsupervised local center of mass (LCM) technique is proposed to segment the spine region and further novel Cobb angle measurement method is proposed for accurate measurement. Validation of the proposed method was carried out on 2D X-ray images from the Saudi Arabian population. Segmentation results were compared with GMM-Based Hidden Markov Random Field (GMM-HMRF) segmentation method based on sensitivity, specificity, and dice score. Based on the findings, it can be observed that our proposed segmentation method provides an overall accuracy of 97.3% whereas GMM-HMRF has an accuracy of 89.19%. Also, the proposed method has a higher dice score of 0.54 compared to GMM-HMRF. To further evaluate the effectiveness of the approach in the Cobb angle measurement, the results were compared with Senior Scoliosis Surgeon at Multispecialty Hospital in Saudi Arabia. The findings indicated that the segmentation of the scoliotic spine was nearly flawless, and the Cobb angle measurements obtained through manual examination by the expert and the algorithm were nearly identical, with a discrepancy of only ± 3 degrees. Our proposed method can pave the way for accurate spinal segmentation and Cobb angle measurement among scoliosis patients by reducing observers’ variability.

## 1. Introduction

Scoliosis is a medical condition characterized by an abnormal curvature of the spine, causing it to curve sideways in an "S" or "C" shape instead of maintaining a straight line [1]. The prevalence of scoliosis can vary globally and is influenced by factors such as age, gender, genetics, and geographic location [2]. According to the Scoliosis Research Society, the estimated prevalence of scoliosis in the general population is approximately 2–3%, while in children and adolescents, it ranges from 2–4% [3]. Previous studies have examined the occurrence of scoliosis in Saudi Arabia [4]. Al Arjani et al. investigated the epidemiological patterns of scoliosis in a specialized hospital in Saudi Arabia, finding that 59% of cases were idiopathic, 7% were secondary to poliomyelitis, and 17% were congenital scoliosis [5]. While scoliosis can affect individuals of any age, it typically develops during childhood or adolescence and can be caused by factors such as genetics, neuromuscular conditions, or idiopathic reasons [6]. Effective treatment and management of scoliosis relies heavily on systematic screening and accurate diagnosis. Screening for scoliosis poses several challenges such as (i) variability in presentation (ii) lack of visible symptoms (iii) age of onset (iv) accessibility to screening (v) observer variability and (vi) cost and resources [7]. These factors can pose challenges, particularly in resource-limited settings or areas with a high prevalence of scoliosis. Addressing these challenges requires a combination of improved awareness, accessible screening programs, standardized diagnostic guidelines, and technological advancements to enhance early detection and accurate diagnosis of scoliosis. The gold standard for diagnosis is the measurement of the Cobb angle, which quantifies the severity of the spinal curvature by assessing the deviation between the two most affected vertebrae [8]. However, accurately tracking the spine and measuring the angle of curvature can be challenging. Traditional measurement methods are susceptible to human errors, leading to inaccurate results [9–11]. Consequently, computer-based methods have been developed to offer more precise and consistent measurements [12].

In medical image analysis, medical image segmentation plays a critical role and finds applications in various areas such as diagnosis, treatment planning, and image-guided interventions [13]. Numerous techniques are available for medical image segmentation, including thresholding, region growing, edge detection, and machine learning-based methods [14,15]. Each technique has its own strengths and limitations, and the selection depends on the specific requirements of the application at hand. In the context of scoliosis diagnosis and treatment, spine segmentation holds significant importance as it enables doctors to evaluate the extent of the condition and formulate suitable treatment strategies [16]. Accurate segmentation of the spine in scoliosis patients is crucial for the accurate diagnosis and treatment of the condition. This process involves precisely identifying the individual vertebrae in the spine, capturing the curvature and alignment accurately. However, achieving accurate segmentation can be particularly challenging, especially when dealing with severe scoliosis or other spinal deformities. In certain situations, manual segmentation may be necessary to ensure precise results. This involves a trained medical professional utilizing specialized software to manually identify the individual vertebrae in the spine. Accurate segmentation of the spine in scoliosis patients is vital for developing appropriate treatment plans and monitoring the progression of the condition over time. While supervised methods are highly effective, they require extensive training datasets with manually labeled images, which can be labor-intensive to produce. On the other hand, unsupervised methods can be employed when training data is unavailable, enabling the segmentation of new images. Aganj et al. introduced the unsupervised local center of mass (LCM) technique [17], an advanced unsupervised machine learning approach utilized for medical image segmentation. This method has demonstrated notable success, particularly in cases where the bones exhibit severe curvature or deformities, as it can adapt automatically without the need for manual intervention [17,18].

Automatic Cobb angle measurement is a computerized method utilized to determine the degree of spinal curvature, specifically for evaluating the severity of scoliosis [19]. Existing methods for Cobb angle measurement can be time-consuming and labor-intensive [20–25]. Earlier researchers have proposed automatic Cobb angle measurement methods that rely on extensive datasets, adding to the labor-intensive nature of the process [26,27]. These methods heavily rely on the training dataset, rather than adapting to new test images. Hence, it is crucial to develop an unsupervised method that can accurately measure the Cobb angle and ease the screening process. In this study, our objective is to employ the unsupervised local center of mass (LCM) technique for accurate segmentation, along with a straightforward Cobb angle measurement technique for screening scoliosis images with varying spinal curvatures.

The proposed work presents a computer vision-based approach for detecting spinal curvature in medical images. The algorithm incorporates various image preprocessing, segmentation, and feature extraction techniques to improve image quality and extract relevant information. The significance of this study lies in its pioneering contribution to the field of scoliosis screening through the utilization of cutting-edge computer vision techniques. By integrating the unsupervised LCM technique for precise image segmentation and a simplified yet effective Cobb angle measurement method, the proposed work addresses critical challenges in the early detection and assessment of spinal curvature abnormalities such as enhanced diagnostic precision, streamlined assessment, potential for early intervention, reduced subjectivity, clinical workflow enhancement etc. In summation, the proposed work’s significance lies in its potential to transform scoliosis screening practices, ultimately leading to improved patient outcomes, reduced healthcare burden, and advancements in the broader field of medical image analysis. The research hypothesis for the proposed work is exploring whether employing the unsupervised LCM technique for segmentation, coupled with the straightforward Cobb angle measurement technique, will lead to a significant enhancement in the accuracy and effectiveness of screening scoliosis images featuring diverse spinal curvatures when compared to conventional methods. Also, the research may lead to finding the key factors influencing the accuracy of the Cobb angle measurement technique in the context of screening scoliosis images, and how do these factors contribute to the overall effectiveness of the screening process.

## 2. Materials and methods

**Fig 1:**
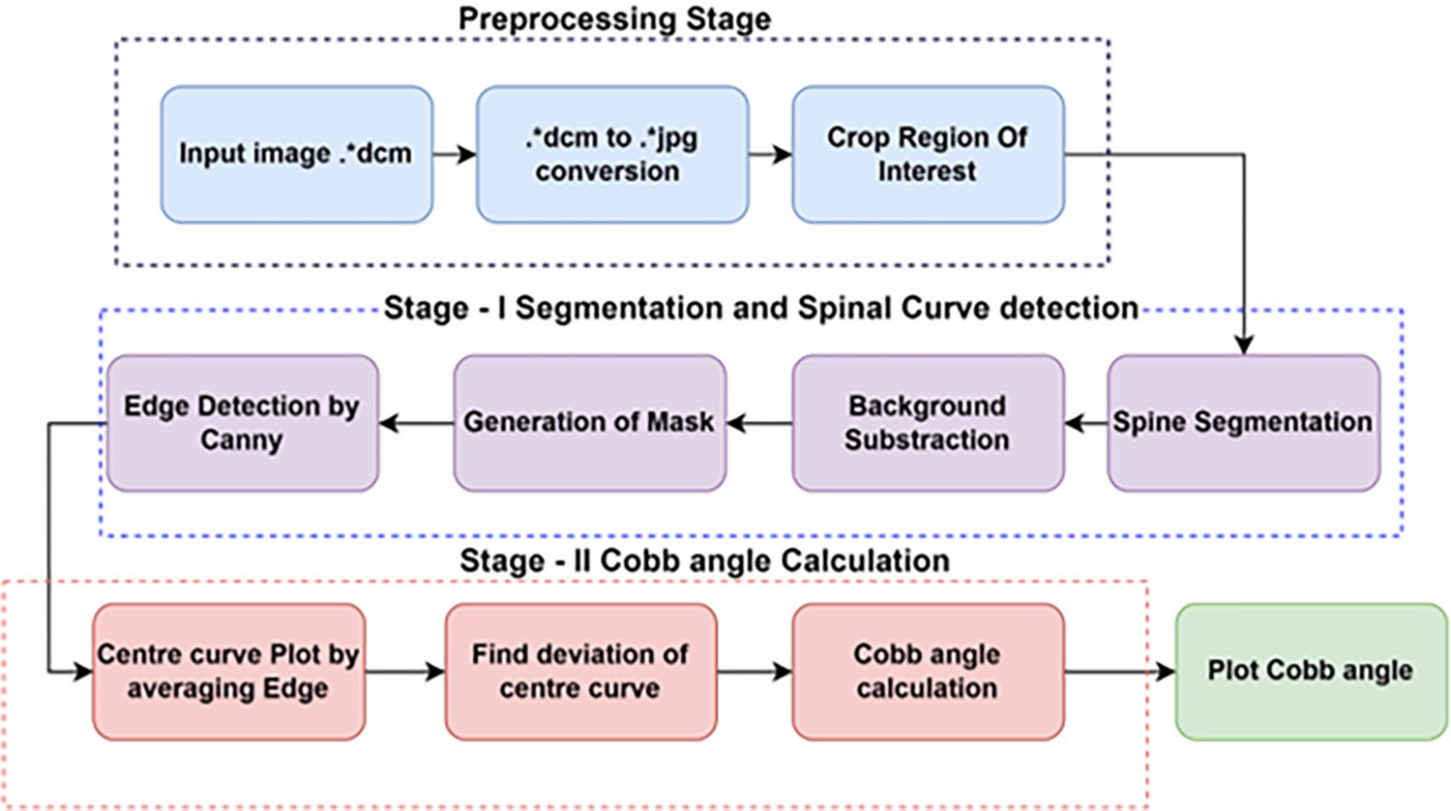
Generalized block diagram of proposed LCM based automatic Cobb angle measurement method.

**Clinical Data Collection:** 50 X-Ray images of Scoliosis digital images were collected from King Fahad Specialist Hospital, King Fahad Medical City (KFMC), Dammam, Saudi Arabia to carry out this research. The Institutional Review Board (IRB) of KFMC has reviewed and approved this study with research protocol (EXT0397) with IRB Log number 22-049E. Scoliosis at various regions of the spine in the age group (20 and 80 years including both Male and Female) were collected during the year 21 February 2022 to 20 February 2023 and the data were authorized to use for research purposes only. The ethics committee waived the need for informed consent. Authors do not have access to information that could identify individual participants during or after data collection. These images were clinically and manually classified by clinical experts. The proposed work presents a computer vision-based approach for detecting spinal curvature in medical images. The algorithm incorporates various image preprocessing, segmentation, and feature extraction techniques to improve image quality and extract relevant information. The generalized block diagram of the proposed LCM based automatic Cobb angle measurement method is shown in Fig 1.

Before commencing the segmentation process, the input scoliosis image undergoes resizing to dimensions of 256 x 256. To ensure the suitability of the image, a filtering operation is performed, ensuring that the image meets the condition of having a dimension less than or equal to 3 within the algorithm. In the pre-processing stage, the focus is on isolating the spine region, determining the region of interest (ROI) for the spine. For enhanced processing efficiency, particular attention is given to the region between the thoracic and lumbar vertebrae in the anteroposterior (AP) view spinal images. This region is referred to as the spine region of interest (spine ROI). Initially, the images are converted into grayscale using a fixed threshold gradient. The histogram of the image is then analysed and Contrast Limited Adaptive Histogram Equalization (CLAHE) is applied to improve the image resolution.

*Preprocessing Stage*: The initial phase of the algorithm involves preprocessing the input data. This includes the essential step of converting DICOM images into the more universally accessible JPG format. By undertaking this conversion, the algorithm ensures that the subsequent processes can be efficiently applied to the images. This preparatory stage sets the foundation for the subsequent operations by transforming the input data into a compatible and manageable format. Following the preprocessing stage, the algorithm advances to the isolation of the region of interest (ROI). This phase entails identifying the specific area within the image that is pertinent to the subsequent analyses. By isolating the ROI, the algorithm narrows its focus to the relevant portion of the image, which is crucial for accurate and efficient processing. This isolation step serves as a critical bridge between preprocessing and the more complex analysis stages.

*Spinal Curve Segmentation and Edge Detection*: The second major stage of the algorithm involves the segmentation of the spinal curve and the detection of its edges. This intricate process employs the Local Centre of Mass technique to perform spinal curve segmentation. Moreover, it incorporates colour intensity information to execute background subtraction, effectively eliminating non-essential elements from the spinal region. The outcome of this stage is an image where the spinal structure is prominently featured, thus facilitating subsequent analyses. Upon achieving the background-subtracted image, the algorithm proceeds to the creation of a binary mask. This mask is produced by converting the modified image into a binary representation, where pixel values are either foreground or background. To determine the edges of the spinal structure in the binary mask, the algorithm employs the Canny edge detection method. This process culminates in the generation of a mask that precisely outlines the spinal structure, essential for the forthcoming computations.

*Cobb Angle Calculation*: In the third stage, the focus shifts to the calculation of the Cobb angle, a pivotal measurement of spinal deviation. This phase involves detecting the center curve, achieved by calculating the average coordinates of the edge pixels obtained from the previously generated edge-detected image. Once the center curve is established, the algorithm applies the Cobb angle formula. This formula yields a numerical representation of the extent of deviation from the center curve, a crucial metric in the medical assessment of spinal conditions. The final step of the algorithm involves the visualization of the calculated Cobb angle. Leveraging the Matplotlib library in Python, the algorithm superimposes the Cobb angle measurement onto the original image. This visual representation provides a clear and concise depiction of the angle’s magnitude and direction, aiding medical professionals in their understanding and interpretation of the spinal condition. This visualization step enhances the algorithm’s practical utility by transforming abstract measurements into comprehensible visual information.

**Fig 2:**
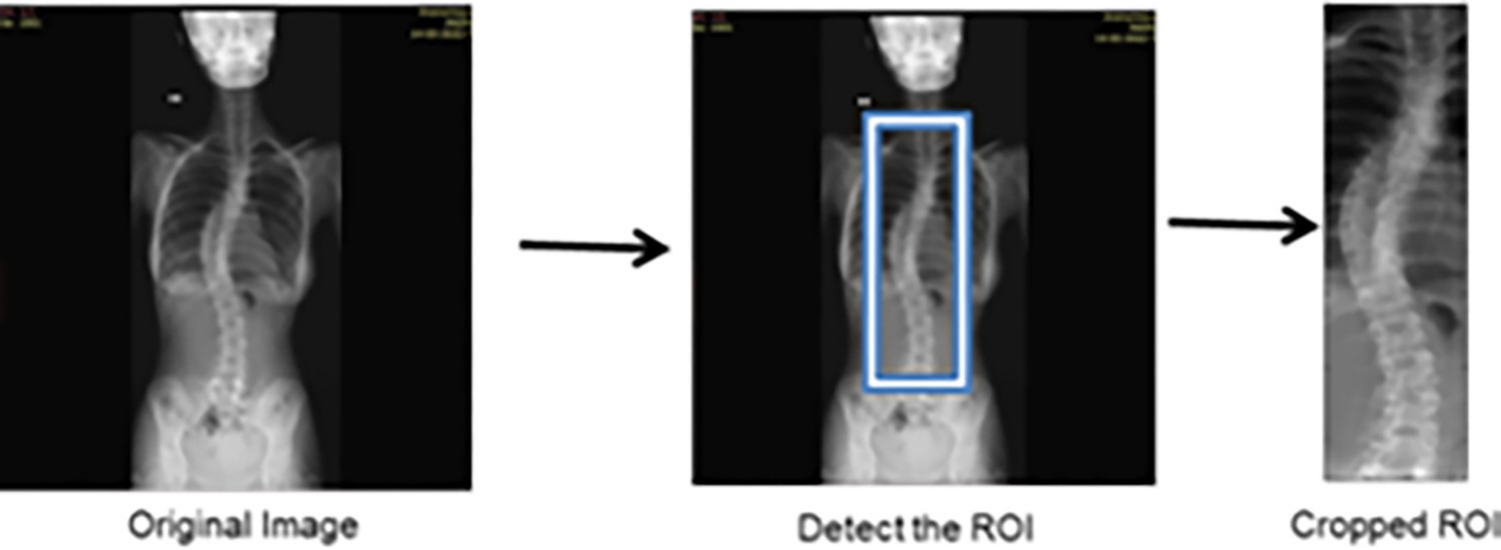
Region of interest cropping from the original image.

The segmentation process employs the LCM (local center of mass) method, and it calculates the center of mass (CM) for the pixels in the image and clusters them into the same or different regions based on their weights. The weights are carefully chosen to minimize computational costs, making the LCM method superior to previous techniques. Overall, the described process encompasses resizing the input image, filtering based on dimension, pre-processing involving spine region isolation and image enhancement, and finally, segmentation using the LCM method with optimized computational efficiency. The segmented images produced using the LCM-based method were compared to the results obtained from the GMM-HMRF algorithm [28,29]. The process of cropping the region of interest (ROI) from the original image is depicted in Fig 2.

### 2.1 Computation of 1D local center of mass

**Fig 3:**
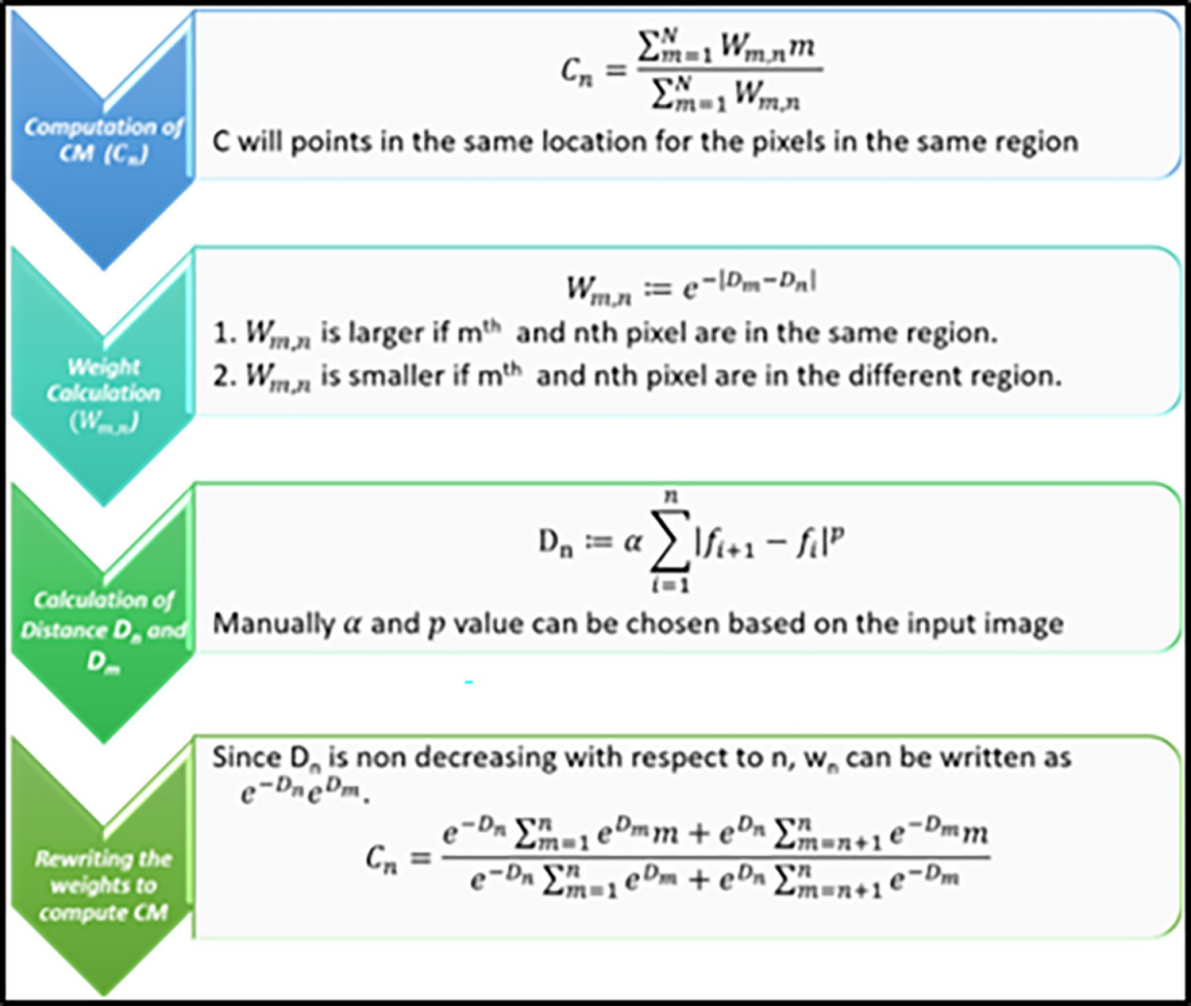
Method of CM computation on an image, Cn = {Cn,_1_, Cn,_2_, …Cn,_4_} of the nth pixel of k = 4iterations.

**Fig 4:**
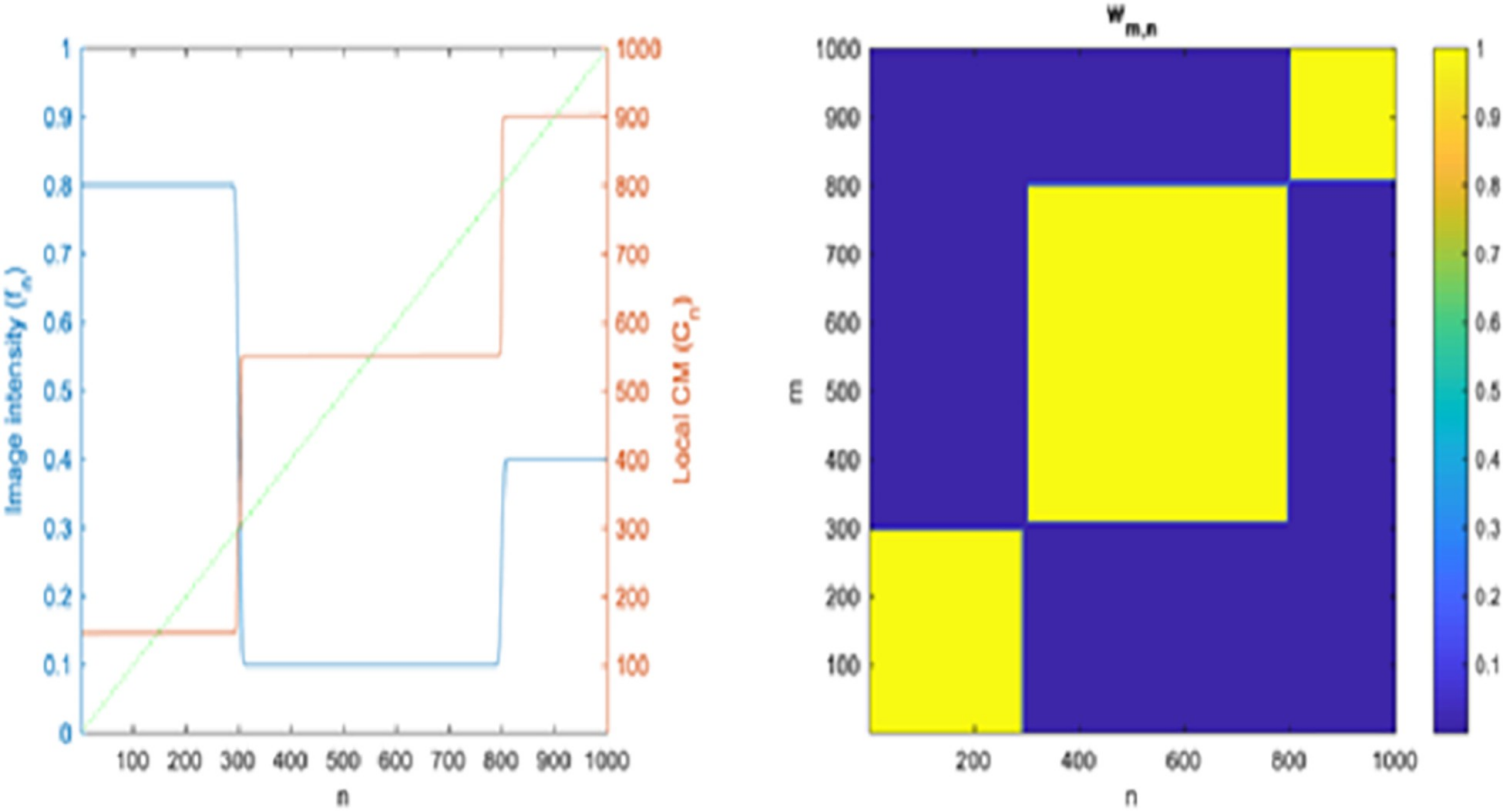
Principle of calculated centre of mass: **(a)** Intensity profile of an image (f_n_, blue) calculated local CM (C_n_, red) and the identity line (n, dotted green) **(b)** Calculated weighting (W_m,n_) of centre of mass.

**Fig 5:**
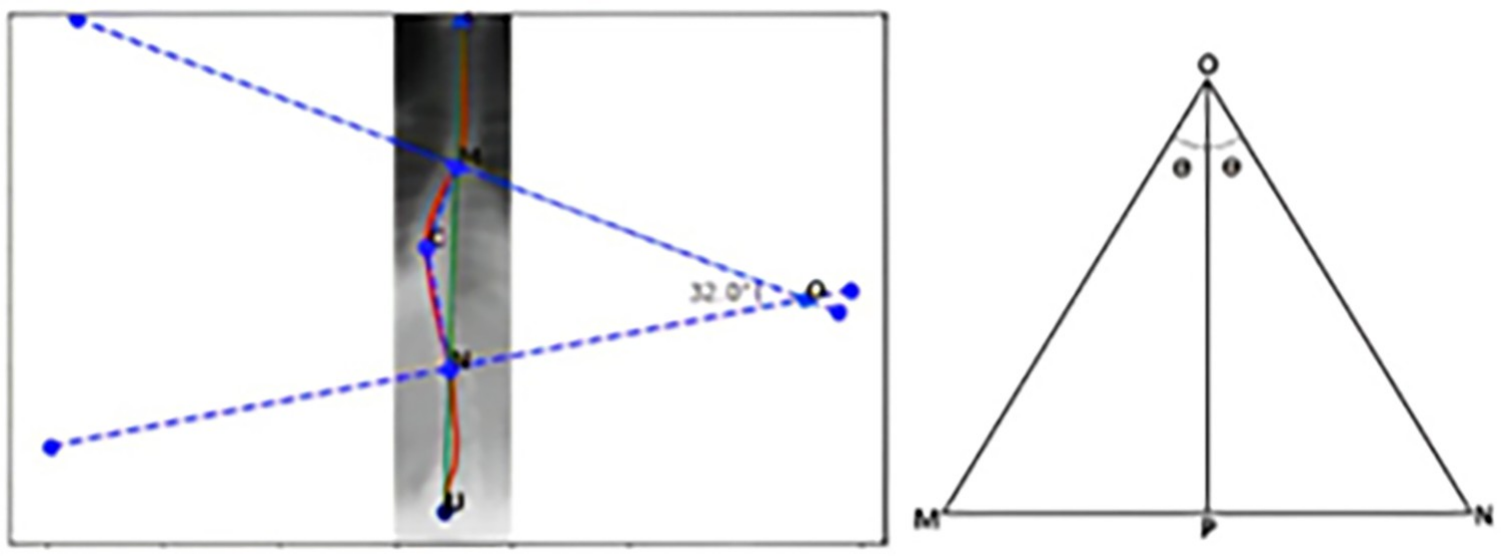
Cobb angle calculation procedure: (a) Centre curve, marking centre point and point of intersection; (b) Cobb angle calculation method on spine image.

The computation of the center of mass (CM) on an image is illustrated in Figs 3 and 4. For a detailed explanation of this CM estimation method, refer to [17]. Fig 5 showcases a visual comparison of image segmentation for scoliosis using the LCM and GMM-HMRF methods, specifically in the thoracic region and thoraco-lumbar regions. Center of mass is a point defined relative to a group of pixels or segment which is calculated with average position of all parts of system, weighted with their intensity values. For a group of pixels with uniform intensity, the center of mass is located at the centroid.

Let f: Ω → ℝ be a 1D discretized image-intensity based sequence of length N, with Ω: = {1, …, N}. Pixels are grouped f into disjoint regions based on the estimated CM of individual pixel’s assumed region, which are called as the LCM, C: Ω →ℝ. Alternatively, the LCM at n, Cn, is identified as the CM of area surrounding the nth pixel. Local CM is calculated as:

$$C_{n}=\frac{\sum_{m=1}^{N}w_{m,n}m}{\sum_{m=1}^{N}w_{m,n}}$$ (1)

Where, W_m,n_ the non-negative weighting work out from f. Then, pixels located in the same area, the C shall point to coarsely the same location (CM of the region), thus assigning those pixels to the same cluster. Hence, the clustering of pixels is done by manipulating the evidence provided in the whole signal, as different to lone its adjoining pixels. Computing C (the entire set {C_n_|n∈ Ω}) is, nevertheless, in overall computationally expensive, costing (N^2^).

***Computational Complexity Reduction*:** To simplify the problem, we need to select a suitable value for w that not only fulfills the mentioned pixel-grouping objective but also effectively reduces the computational cost of C. By adopting the following aproach for w, we can substantially minimize the computation cost of C to approximately Ο(N).

$$w_{m,n}≔e^{-|D_{m}-D_{n}|},$$ (2)

Where,

$$D_{n}≔\alpha \overset{n}{\underset{i=1}{\sum }}{\left|f_{i+1}-f_{i}\right|}^{p},$$ (3)

with manually chosen α, p > 0 (We define f _N +1_ = f _N_ for ease of notation). As the signal edges between the m^th^ and n^th^ pixels become increasingly pronounced, the absolute difference, |D_m_ − D_n_|, grows larger, resulting in a smaller w_m,n._ This behavior signifies that these two pixels belong to distinct regions. Conversely, when there are few noticeable edges between the m^th^ and n^th^ pixels, w_m,n_ assumes a larger value, indicating that both pixels are within the same region. Fig 4A illustrates this concept with a 1D signal f_n_ (left, represented by the blue curve) and its corresponding w_m,n_ computation (right). It’s worth noting that Eq (3) can be readily extended to multichannel images, such as RGB, by independently calculating the sum for each channel and aggregating the results to obtain a composite D_n_. When calculating C, we can take advantage of the fact that D_n_ increases monotonically with respect to n. Consequently, w_m,n_ can be expressed as as e^-D n^ e ^D m^ for m≤n and e^D n^ e ^-D m^ for m>n. This allows us to expand Eq (1) to compute the components of ’C’ as follows:

$$C_{n}=\frac{e^{-D_{n}}\sum_{m=1}^{n}e^{D_{m}}m+e^{D_{n}}\sum_{m=n+1}^{N}e^{-D_{m}}m}{e^{-D_{n}}\sum_{m=1}^{n}e^{D_{m}}+e^{D_{n}}\sum_{m=n+1}^{N}e^{-D_{m}}}.$$ (4)

Given that all the sums in Eqs (3) and (4) can be pre-computed recursively in Ο(N) and stored for all n, the entire C can now be computed efficiently and non-iteratively in Ο(N). As expected, C_n_ is piecewise constant. and its value points to the CM of each interval in the signal (e.g., it intersects the identity line at the centers of the intervals).

### 2.2 Cobb angle calculation

**Fig 6:**
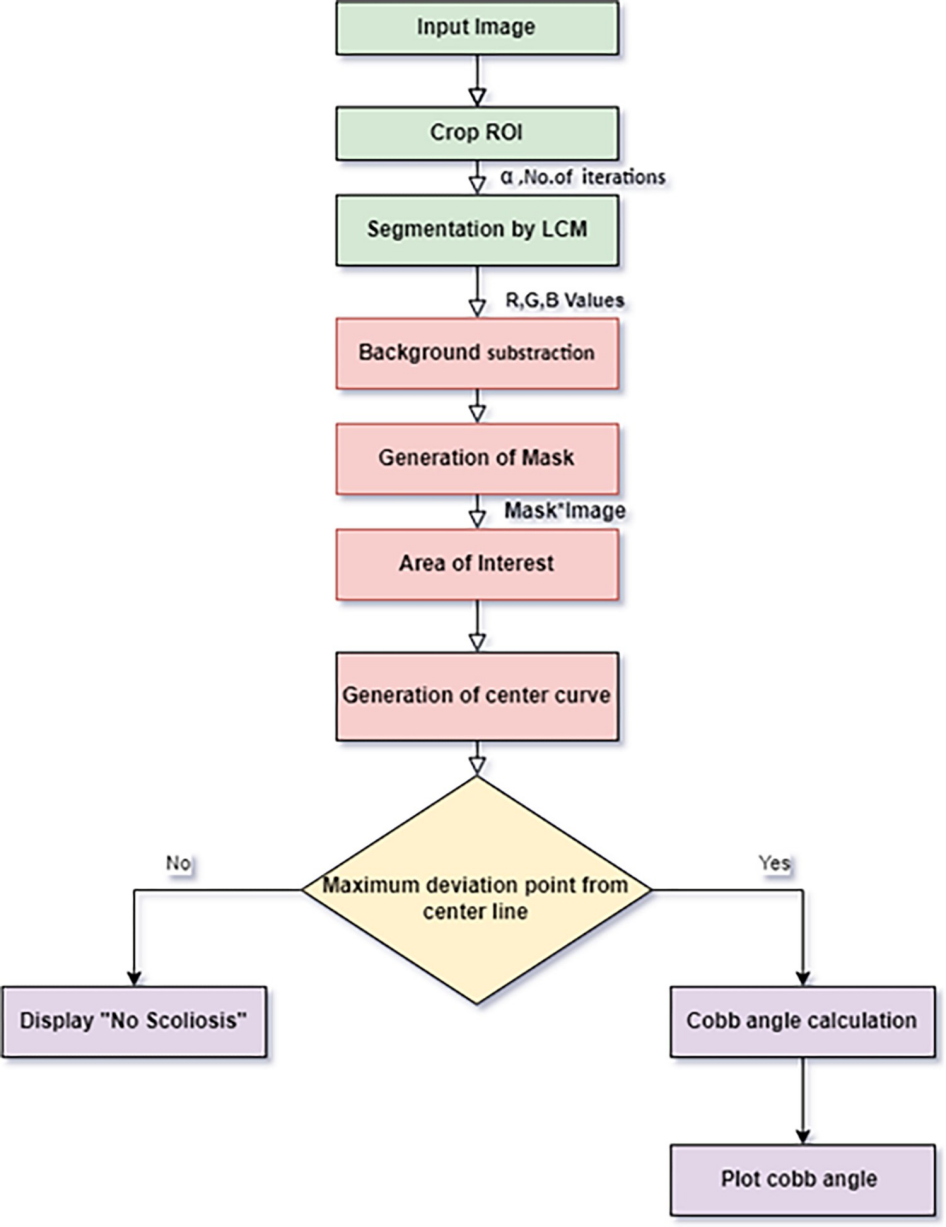
Algorithm flow chart to find Cobb angle for image directory.

In this study, a straightforward and innovative technique for measuring the Cobb angle was developed. The process involves several steps using Python and matplotlib after obtaining the image’s center curve through Canny edge detection and averaging techniques. The entire Cobb angle measurement is conducted with respect to the original image. To locate the end coordinates of the curve depicted in Fig 5, it is necessary to identify the upper and lower points, denoted as L and U, respectively. Once these points are determined, a line connecting them is drawn, intersecting the center curve at points M and N from the top and bottom, respectively. In Fig 4A, the center curve is highlighted in red, while the line connecting points L and U is presented in green. After locating the intersection points M and N as shown in the diagram, point C is determined in the image by finding the minimum X-coordinate of the center curve. This point corresponds to the maximum deviation of the curve from its mean position. To visualize this deviation, lines are drawn between point M and C, and point N and C, represented by blue dotted lines in Fig 5A. Subsequently, lines perpendicular to MC and NC are drawn from points M and N, respectively, as depicted in Fig 5B. These perpendicular lines are extended until they intersect at point O. According to the law of trigonometry,

$$from\;△MOP,\;Ɵ={tan}^{-1}\;\frac{OP}{MP}\;and\;2Ɵ\;gives\;Cobb\;angle$$ (5)

where MON represents the Cobb angle in Fig 6B. The algorithm developed for Cobb angle measurement is implemented using the Python 3.9 64-bit compiler, and the resulting angle is calculated. The obtained results are plotted using the matplotlib library. In Fig 5A, the calculated angle is displayed near point O of the image. This allows for a clear visualization of the Cobb angle for monitoring purposes. The algorithm not only calculates the angle but also provides a visual plot for the user’s convenience. To summarize, this study introduces a computer-based approach for accurately measuring the Cobb angle from X-ray images of scoliosis. The detailed results obtained from this methodology will be presented in the subsequent section.

A comprehensive flowchart illustrating the entire methodology and the corresponding algorithm developed in this study is presented in Fig 6. This flowchart offers a visual representation of the Cobb angle calculation process, enabling users to monitor the results effectively.

## 3. Results

**Fig 7:**
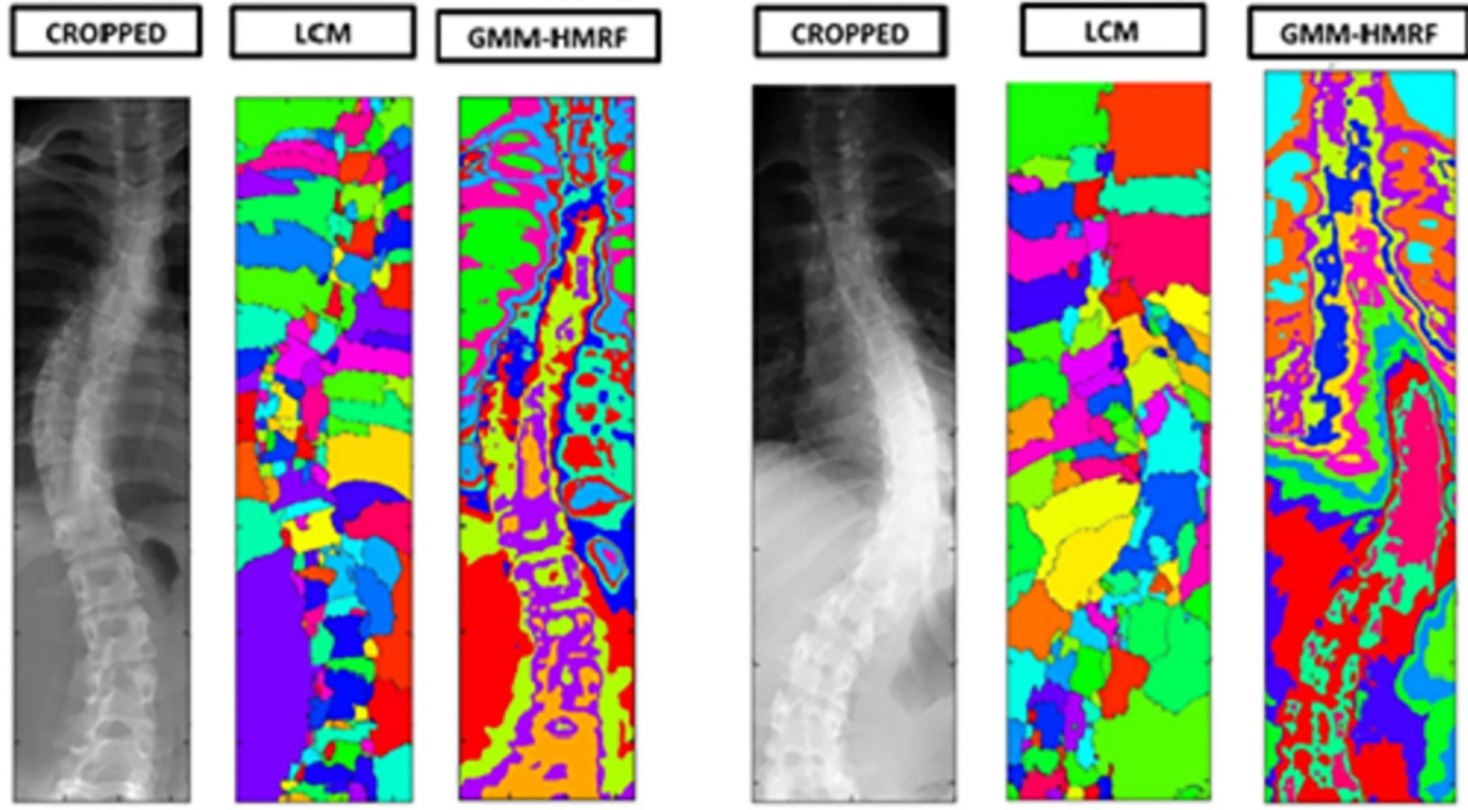
X-ray Image Segmentation of Scoliosis in (a) Thoracic region (b) Thoraco-Lumbar region.

The scoliosis images were preprocessed using standard techniques, including grayscale conversion and noise reduction. We first evaluated the two (proposed LCM-based and GMM-HMRF) methods on a sample image dataset containing different scoliosis cases (Fig 7), with dimensions of 493x2077 pixels. Here, images were initially cropped for ROI by horizontal and vertical histograms and then normalized by converting it into binary format. We computed the local CMs in K = 180 orientations with an angular resolution of 1°. Fig 7A shows segmentation by the proposed LCM method with optimal values of α* = 2000, and Fig 7B represents the results of GMM-HMRF method with t * = 750 phase-1 iterations (1,000 total iterations) and found the optimal value of r* = 5 and g* = 1. The dice score index is used for quantitative analysis of segmented images from the greyscale by comparing the binary image to its ground truth. The dice score of 1 indicates the ideal segmentation whereas 0 indicates poor. The quantitative analysis of the proposed LCM image segmentation is performed in terms of accuracy, sensitivity, and specificity to evaluate the effectiveness of segmentation edges. We tested the above-mentioned procedure with several hardware distributions. Hence, to compare the computational time of two methods in the same system, we executed the final algorithms on a laptop with two Intel Core i5^®^ 2.40 GHz processors 4GB RAM.

**Fig 8:**
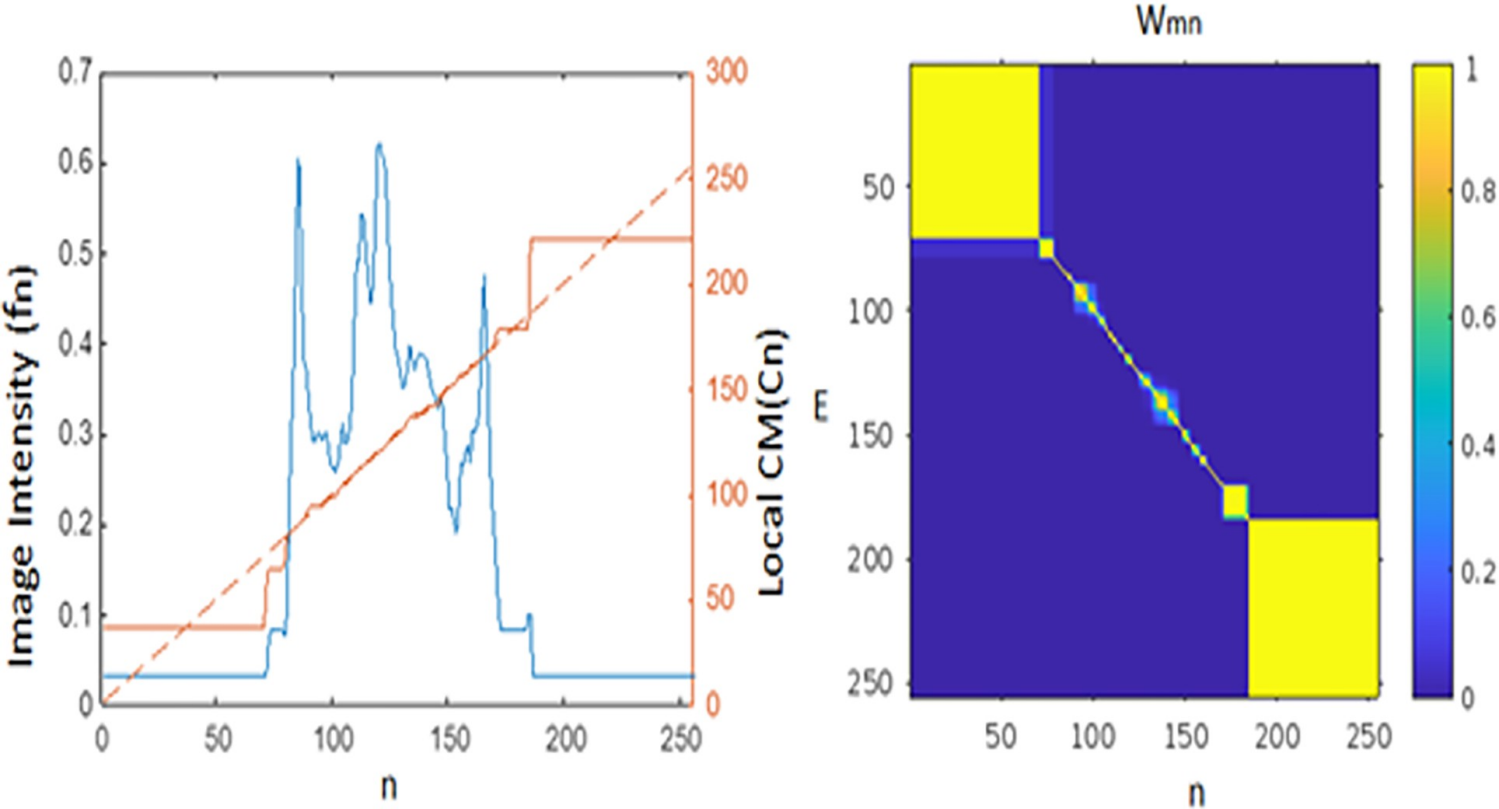
Left: Intensity profile of the horizontal blue line in Fig 7 (top, left), indicated in blue. Additionally, the local CM in red and the identity line as a dotted green line are presented. Right: Depiction of the weighting (w_m,n_).

The 1D image intensity profile along the horizontal blue line shown in Fig 7 (top, left) is visualized in Fig 8 as the blue curve. Additionally, the local Contrast Measure (CM) for this 1D signal is computed using Eq (4) with α = 200 and depicted as the red curve. The green dotted line represents the identity function serving as a reference. Notably, the red curve representing the local CM appears to exhibit an almost piecewise constant pattern, with its values signifying the centers of the 1D intervals. Based on the experiment we selected an optimal value of both α and iterations which is provided in Table 1.

**Table 1:** Optimal values of the parameters chosen for the two methods.

Parameters	LCM	GMM-HMRF
CV Dice	0.51±0.04	0.54±0.01
Optimal parameters	α* = 2000±500 & t* = 1000±250	r* = 5±0.5; g* = 1±0 t = 30, T = 30
Tested range of parameters	α: 100~5000 t: 10~5000	r: 2~20; g: 1~5 t = 5~100, T = 5~100
CPU Runtime	5400±600s (1.5hr±10mins)	1800±300s (0.5hr±5mins)

Commonly employed metrics for assessing the efficacy of segmentation methods include accuracy, sensitivity, and specificity. These metrics offer a glimpse into the performance of a segmentation algorithm across various facets [30]. Performance of the proposed LCM segmentation was evaluated in terms of accuracy, sensitivity, and specificity for its effectiveness of segmentation edges. Accuracy is a measure of the overall correctness of the segmentation results and is calculated as below,

$$Accuracy=\frac{Number\;of\;correctly\;classified\;pixels\;in\;RoI}{Total\;number\;of\;pixels\;in\;RoI}$$ (6)

Sensitivity measures the ability of a segmentation method to correctly identify positive instances and is calculated as below,

$$Sensitivity=\frac{True\;Positive}{True\;Positives+False\;Negatives}$$ (7)

Specificity measures the ability of a segmentation method to correctly identify negative instances and is calculated as below,

$$Specificity=\frac{True\;Negative}{True\;Negatives+False\;Positives}$$ (8)

The proposed LCM method is compared with GMM-HMRF, and parameters are obtained and compared side by side as shown in Table 2. The confusion matric overlay is generated for both GMM-HMRF and LCM method and compared. Through qualitative and quantitative (accuracy &dice score) validation, it is proven that the proposed LCM method performed better than existing unsupervised segmentation GMM-HMRF method. The LCM algorithm is more flexible with parameters like α, and iterations compared to other segmentation methods. The LCM method generally produced less over-segmented results than the GMM-HMRF which prevent errors due to grey scale exposure.

**Table 2:** Quantitative analysis of LCM and GMM-HMRF segmentation methods.

Method	Sensitivity^1^	Specificity^1^	Accuracy^1^	Dice score^2^
LCM	90.5	98.4	97.3	0.54
GMM-HMRF	88.24	90	89.19	0.51

^1^ Expressed in terms of % and ^2^ as a ratio of two times the spatial overlap area to the total number of pixels in segmented and ground truth images.

**Fig 9:**
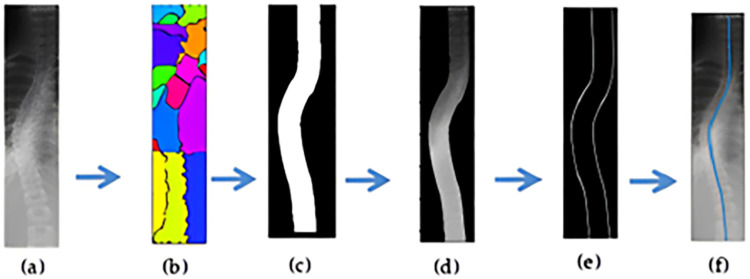
Background subtraction process to separate spinal region: (**a**) input spine image; (**b**) LCM segmented image with predefined colors; (**c**) binary matrix of the mask; (d) cropped image after background subtraction; (e) after canny edge detection; (f) center line marking.

**Fig 10:**
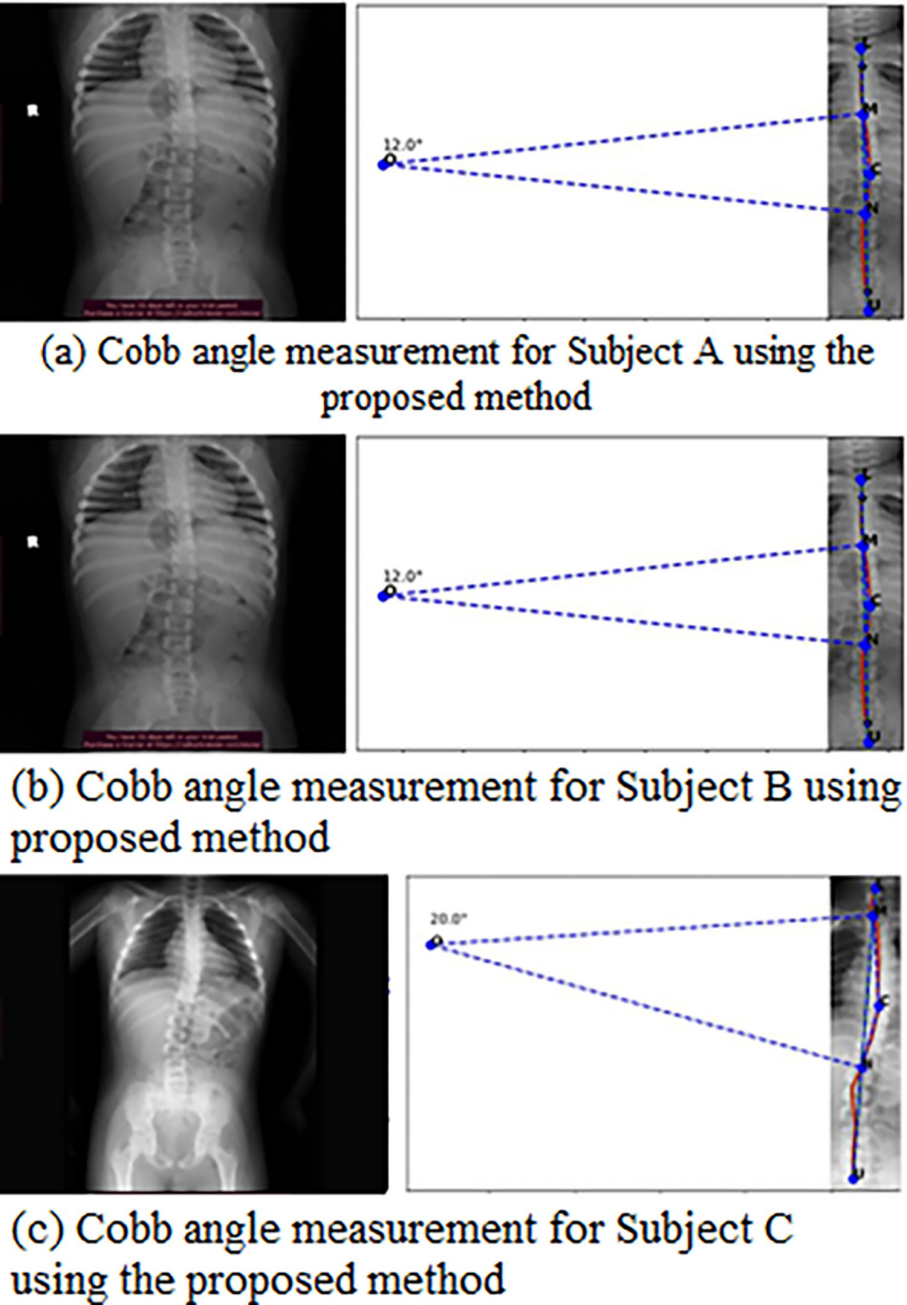
Measurement of Cobb angle using the proposed method on various scoliosis cases.

**Fig 11:**
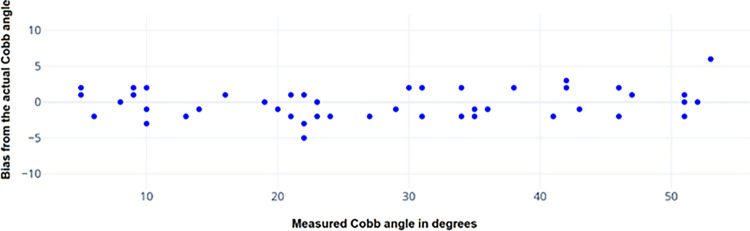
The bias of measured Cobb angle from the actual angle obtained from ground truth images for 50 subjects.

After LCM segmentation, the spine was cropped, and the color subtraction technique was used to create a binary mask. The spinal curvature was traced by finding the coordinates of the binary mask edges using the Canny edge detection algorithm [23]. The method of separating the spine region and finding the center curve of the spine is described in Fig 8. Fig 9A was the input scoliotic spine image and Fig 9B shows the segmented image with predefined colors for the spinal region and background area. To generate the mask, the RGB values were specified in the background colour of the segmented image. After applying background subtraction, a binary matrix of the mask is obtained, as shown in Fig 8C. Finally, the complete background sub-traction image is generated by multiplying the binary matrix with the cropped image matrix, as shown in Fig 9D. The next step is to find the center curve of the spine. This is achieved by extracting the coordinates of the edges in the background-subtracted image, which correspond to the center line of the spine. To obtain the center spinal curve, we apply canny edge detection to the ROI image, as shown in Fig 8E. The resulting edge coordinates are stored as a matrix, and the center line is calculated by taking the average of the two edge coordinate value matrices. This center line based on the average values is then plotted over the original cropped image, as shown in Fig 9F. This curve is subsequently used to determine the Cobb angle for the given spine image. The Cobb angle is calculated based on the maximum deviation point from the vertical axis, if there is not much deviation of the curve, then the algorithm automatically labels the image as no scoliosis case and continues to the next images. Finally, the algorithm writes the resulting images in the respective directories based on the decision. Fig 10 shows the measurement of Cobb angle using the proposed method on three various scoliosis cases and Fig 11 shows the bias of the measured angle with respect to ground truth images.

## 4. Discussion

The proposed methodology offers enhanced accuracy and consistency in measuring the Cobb angle, thereby holding promise for advancing the diagnosis and treatment of scoliosis. The introduction of the LCM technique for image segmentation enhances the accuracy of identifying relevant spinal regions in medical images leads to diagnostic precision. This automated approach reduces the potential for human error, variability, and bias that often accompany manual segmentation methods. The resulting segmented images provide a solid foundation for subsequent analysis. The incorporation of a straightforward Cobb angle measurement method streamlines the assessment of spinal curvature severity. By directly quantifying the Cobb angle from segmented images, the proposed work offers a time-efficient and standardized approach, empowering clinicians to make informed decisions promptly.

The implementation of threading was not incorporated into the codes. However, it is worth noting that the algorithm’s complexity led to phase-2 iterations taking approximately seven times longer to execute compared to phase-1 iterations. In the experiment, we executed the algorithm with a total of 1000 iterations, including 250 phase-2 iterations. To further optimize the runtime, the number of phase-2 iterations can be reduced based on the desired segmentation quality. Additionally, employing a smaller number of orientations (K) and executing the algorithm on a high-end GPU can contribute to runtime reduction. Furthermore, it was observed that increasing the values of α and iterations resulted in over-segmentation, but this can be mitigated by selecting optimal parameter values as recommended by Aganj et al [17]. To assess the efficacy of the approach, a comparison was made between the results obtained and the assessments made by senior scoliosis surgeons at a multi-specialty hospital in Saudi Arabia. The findings revealed that the LCM segmentation of the vertebrae was remarkably accurate, closely resembling the ground truth images with a dice coefficient of 0.542, surpassing the dice score value of 0.512 obtained by GMM-HMRF. The LCM segmentation exhibited an overall accuracy of 97.3%, whereas the GMM-HMRF achieved an accuracy of 89.19%. It was observed that the GMM-HMRF model placed greater emphasis on image intensity rather than the geometry of a segment, resulting in segmentation that was more tissue-oriented rather than organ-specific. The GMM-HMRF model, which assigns a single Gaussian to each detected linked tissue type, proved to be less effective in segmenting specific bones [17] (Fig 7).

Moreover, the Cobb angle measurements were obtained through manual examination by senior scoliosis surgeons at the multi-specialty hospital in Saudi Arabia. The proposed novel method exhibited precise calculation of the Cobb angle in scoliosis, surpassing the performance of other existing methods (Fig 11). The proposed method effectively tackles the issue of improper edges in the spinal vertebral mask by implementing the center curve smoothing technique and thus reducing observers’ variability. The algorithm estimates Cobb angles with a minimal bias of only ± 3 degrees, which aligns with the findings of recent studies on automatic Cobb angle measurement [31,32]. Notably, the proposed LCM-based method does not rely on pretrained models or weights, eliminating the need for time-consuming training, validation, and testing steps typically seen in CNN-based methods [32]. This feature enables the calculation of Cobb angles in diverse scoliosis cases using segmented masks. Rapid and reliable scoliosis screening is pivotal for timely therapeutic interventions. The proposed approach’s accuracy and efficiency hold the promise of identifying spinal irregularities at an early stage, allowing for proactive interventions, and minimizing potential complications [17,18]. The proposed automation of segmentation and Cobb angle measurement reduces subjectivity in diagnosis, leading to increased consistency and reliability of results across different medical practitioners and settings. Integrating computer vision techniques into clinical practice has the potential to enhance workflow efficiency. By automating time-consuming tasks such as segmentation and angle measurement, medical professionals can allocate more time to patient care and decision-making. In the era of telemedicine, automated image analysis techniques can facilitate remote assessments. The proposed approach could serve as a valuable tool for remotely evaluating spinal curvature, thereby expanding access to healthcare services. However, this study has certain limitations that are: (i) The accuracy of the proposed method relies heavily on the quality of segmentation achieved by the Local Centre of Mass (LCM) method. Therefore, selecting appropriate parameters for LCM becomes crucial for the success of our approach. (ii) The proposed method involves a higher runtime, resulting in increased computation costs. It necessitates a higher-end system with GPU and processors, adding to the overall cost of computation. However, by reducing phase-2 iterations and the number of orientations, considerable results can still be obtained while minimizing the runtime and computational requirements. (iii) The utilization of intensive graphics in the proposed method requires a system with capable graphics cards, which further adds to the cost of computation. Additionally, the LCM segmentation method is time-consuming compared to other approaches. However, by executing algorithms with lower numbers of orientations (K) and iterations, the computational cost and runtime can be minimized, while still yielding reasonable results.

## 5. Conclusions

In this investigation, an unsupervised LCM technique is introduced for the segmentation of the spinal region, coupled with an innovative approach for the precise measurement of the Cobb angle. The proposed methodology underwent validation using 2D X-ray images from the Saudi Arabian population. Segmentation outcomes were systematically compared with the GMM-HMRF segmentation method, assessing sensitivity, specificity, and dice score metrics. Our segmentation approach showcased an overall accuracy of 97.3%, outperforming the 89.19% accuracy achieved by GMM-HMRF. Additionally, our method exhibited a superior dice score of 0.54 compared to GMM-HMRF. The introduced approach successfully addresses concerns related to inaccurate edges in the spinal vertebral mask by incorporating the center curve smoothing technique, thereby minimizing variability among observers. The proposed algorithm accurately estimates Cobb angles with a minimal bias, showing a narrow range of only ± 3 degrees. Future work will focus on developing an algorithm that autonomously optimizes parameters based on input images, enhancing segmentation quality crucial for accurate Cobb angle calculations. The method relies heavily on precise image segmentation, prompting exploration of advanced techniques for improved accuracy. For pre-segmented images, maintaining fixed labels or updating them during iterations will benefit semi-supervised segmentation. Ultimate goal is to transform the algorithm into a web application for easy image upload and Cobb angle results retrieval. Additionally, it is aimed to create an Android app for real-time Cobb angle calculations from digital X-ray images. In summary, this methodology propels the field of medical image analysis, promoting synergy between computer science and medicine. It lays the groundwork for continued research, refining the applications of computer vision in diverse medical imaging challenges and potentially resulting in advanced algorithms for comprehensive spinal assessment. Furthermore, this research has the capacity to transform scoliosis screening, improving patient outcomes, lessening the healthcare burden, and pushing the boundaries of medical image analysis.
